# P1NP and β-CTX-1 Responses to a Prolonged, Continuous Running Bout in Young Healthy Adult Males: A Systematic Review with Individual Participant Data Meta-analysis

**DOI:** 10.1186/s40798-023-00628-x

**Published:** 2023-09-19

**Authors:** Rita Civil, Eimear Dolan, Paul A. Swinton, Lívia Santos, Ian Varley, Philip J. Atherton, Kirsty J. Elliott-Sale, Craig Sale

**Affiliations:** 1 School of Sport, Exercise and Rehabilitation Sciences, University of Birmingham, Birmingham, United Kingdom; 2 Musculoskeletal Physiology Research Group, Sport, Health and Performance Enhancement (SHAPE) Research Centre, School of Science and Technology, Nottingham Trent University, Nottingham, United Kingdom; 3 Applied Physiology and Nutrition Research Group, School of Physical Education and Sport, Rheumatology Division; Faculdade de Medicina FMUSP, Universidade de Sao Paulo, São Paulo, Brazil; 4 School of Health Sciences, Robert Gordon University, Aberdeen, UK; 5 Centre of Metabolism, Ageing and Physiology (CMAP), MRC-Versus Arthritis Centre of Excellence for Musculoskeletal Ageing Research, Nottingham NIHR Biomedical Research Centre, School of Medicine, University of Nottingham, Derby, United Kingdom; 6 Department of Sport and Exercise Sciences, Institute of Sport, Manchester Metropolitan University, Manchester, United Kingdom

**Keywords:** Bone remodelling, Bone markers, Exercise, Running, Inter-individual variability, Proportion of response

## Abstract

**Background:**

Circulating biomarkers of bone formation and resorption are widely used in exercise metabolism research, but their responses to exercise are not clear. This study aimed to quantify group responses and inter-individual variability of P1NP and β-CTX-1 after prolonged, continuous running (60–120 min at 65–75% V̇O_2max_) in young healthy adult males using individual participant data (IPD) meta-analysis.

**Methods:**

The protocol was designed following PRISMA-IPD guidelines and was pre-registered on the Open Science Framework prior to implementation (https://osf.io/y69nd). Changes in P1NP and β-CTX-1 relative to baseline were measured during, immediately after, and in the hours and days following exercise. Typical hourly and daily variations were estimated from P1NP and β-CTX-1 changes relative to baseline in non-exercise (control) conditions. Group responses and inter-individual variability were quantified with estimates of the mean and standard deviation of the difference, and the proportion of participants exhibiting an increased response. Models were conducted within a Bayesian framework with random intercepts to account for systematic variation across studies.

**Results:**

P1NP levels increased during and immediately after running, when the proportion of response was close to 100% (75% CrI: 99 to 100%). P1NP levels returned to baseline levels within 1 h and over the next 4 days, showing comparable mean and standard deviation of the difference with typical hourly (0.1 ± 7.6 ng·mL^−1^) and daily (− 0.4 ± 5.7 ng·mL^−1^) variation values. β-CTX-1 levels decreased during and up to 4 h after running with distributions comparable to typical hourly variation (− 0.13 ± 0.11 ng·mL^−1^). There was no evidence of changes in β-CTX-1 levels during the 4 days after the running bout, when distributions were also similar between the running data and typical daily variation (− 0.03 ± 0.10 ng·mL^−1^).

**Conclusion:**

Transient increases in P1NP were likely biological artefacts (e.g., connective tissue leakage) and not reflective of bone formation. Comparable small decreases in β-CTX-1 identified in both control and running data, suggested that these changes were due to the markers’ circadian rhythm and not the running intervention. Hence, prolonged continuous treadmill running did not elicit bone responses, as determined by P1NP and β-CTX-1, in this population.

**Supplementary Information:**

The online version contains supplementary material available at 10.1186/s40798-023-00628-x.

## Key Points

- It is unclear whether a single running bout produces bone adaptations, but these potential responses were not captured by bone (re)modelling markers P1NP and β-CTX-1.
- There is a need for studies that investigate the acute responses of bone (re)modelling markers to different types of exercise interventions and across different populations, which include a control (non-exercise) group.

**Supplementary Information:**

The online version contains supplementary material available at 10.1186/s40798-023-00628-x.

## Introduction

Weight-bearing exercise is generally considered to be beneficial for bone health and is associated with long-term (i.e., months, years) improvements in bone mineral density (BMD) and bone architecture, particularly at load bearing sites [1–4]. Although the best exercise regimen (i.e., type, intensity, duration, and frequency) to optimise bone responses is still not well defined, research suggests that dynamic, high-impact, rapid, multi-directional movement patterns and unaccustomed loads, with a sufficient load intensity, are likely to produce the largest osteogenic stimulus [5–8]. The effects of endurance running exercise on bone are interesting because, although running produces greater gravitational loading compared to other low-impact activities, such as cycling [9], it also has a repetitive loading cycle and has been associated with a relatively high prevalence of stress fracture injury [10, 11]. Low BMD is prevalent in endurance runners, particularly at non-loaded sites [12], and it seems that beneficial effects of mechanical loading may not counteract the potential negative influences associated with endurance exercise [11], such as micro-damage accumulation and low energy availability [13].

Examining the dynamic bone response to acute running exercise bouts is a logical approach to further investigate the effects of this exercise type on bone, which can be done by measuring changes in bone (re)modelling markers, measured in blood, before and after a running intervention. Almost all studies that have included these measurements, however, were not designed to directly answer this question and did not include a control (non-exercise) group, which makes it difficult to separate running-induced responses from circadian variation [14]. Furthermore, the results from the few studies that have included a running intervention and a control group are inconsistent. Two studies reported no significant differences in bone formation marker P1NP levels 1-24 h hours after an intermittent [15] or a continuous [16] bout of running compared to a non-exercise control condition, but Alkahtani et al. [17] reported increases in P1NP immediately and 24 h after intermittent running. In terms of bone resorption, increases in β-CTX-1 have been shown 1 h, but not 24 h, after intermittent running [15] and 24-96 h after continuous running [16]. Potential explanations for these discrepant results include differences in exercise regimen (i.e., duration, intensity, intermittent/continuous) and measurement error (i.e., instrumentation and biological noise), and lack of standardisation of factors such as sleep, diet, physical activity prior to and following the running bout. It is also possible that different individuals respond differently to the exercise intervention itself (inter-individual variability).

The extent of inter-individual variation in the bone biomarker response to prolonged continuous running is unknown. The estimation of the typical variation in observed scores derived from measurement or biological noise can be quantified through the variation in scores in control conditions (by including a control group) [18]. For example, when investigating the responses of bone (re)modelling markers to an exercise intervention, the estimation of typical variation would allow quantification of the degree to which the observed changes were affected by factors external to the intervention itself, such as circadian rhythms, and, therefore, would allow quantification of the degree to which the intervention itself may contribute toward the observed variation. Whilst obtaining accurate estimates of these variability assessments is difficult for single studies, individual participant data (IPD) meta-analytic approaches provide better estimates of mean responses with larger sample sizes and allow for the assessment of effects at the participant level by using the raw data from selected studies [19, 20], and, thus, can determine inter-individual variability.

To better understand bone responses to acute bouts of running, the aims of this study were to (i) evaluate the mean responses of P1NP and β-CTX-1 to a prolonged, continuous running exercise bout in young healthy adult males, (ii) estimate the inter-individual variability in bone (re)modelling marker responses, and (iii) determine to what degree any inter-individual variability was associated with the prolonged, continuous running bout itself (herein termed the intervention response), versus those related to external factors such as circadian variation.

## Methods

The protocol for this review included all items described in the checklist of Preferred Reporting Items for Systematic Review and Meta-Analysis of Individual Participant Data (PRISMA-IPD) [20, 21]. The protocol for this review was pre-registered on the Open Science Framework prior to implementation (https://osf.io/y69nd).

### Updates on the Pre-registered Protocol

In the pre-registered protocol, a combined approach of aggregate data and individual participant data meta-analyses was proposed. Because individual participant data were obtained from all running studies, however, the aggregate analysis was deemed unnecessary for the purpose of this investigation. The pre-registered protocol indicated that the statistical model would include the estimation of variability ratio (ratio of standard deviation of inter-individual difference scores relative to measurement error values); this estimation was not included in the statistical approach due to the fact that measurement error values were often as large as variation in the intervention.

### Eligibility Criteria

The PICOS (Population, Intervention, Comparator, Outcomes and Study Design) approach was used to guide the determination of eligibility criteria for this study.

#### Population

Studies that included young (18–35 years old), healthy (i.e., non-smokers, injury free and not taking medication from any condition known to affect bone metabolism), active males (V̇O_2max_ ~ 50 mL·kg^−1^·min^−1^) were considered for inclusion. Differences in training status are unlikely to influence the responses of bone (re)modelling markers after a running exercise bout [16] and, therefore, participants included young healthy males who were active (i.e., recreationally) or endurance trained (e.g., runners, triathletes). Only male participants were included because most studies in this area have focused upon young, healthy, adult male populations. Studies in healthy active females are lacking on this topic, a disparity that is considered in the discussion.

#### Intervention

The term ‘intervention’ was taken to mean a prolonged, continuous running bout, regardless of whether or not this was the focus of the original studies from which the data were extracted. Studies were considered for inclusion if they included blood sample collections at baseline, before, during, and after prolonged, continuous treadmill running at an intensity of ≥ 65% V̇O_2max_ and with a duration of 60–120 min. In order to reduce variation due to circadian rhythms [14, 22] and feeding [23–25], studies were only included if they were conducted in the morning with a baseline sample collected after an overnight fast and the rest of the samples collected in a fasted state or after consuming a non-caloric placebo. Studies were only included in this review if they involved a continuous treadmill running-based exercise bout to control for mechanical loading across studies.

#### Comparator

Bone (re)modelling marker responses to running were measured by comparing changes in markers during and post-running (i.e., from blood samples taken during and in the hours and days after the running bout) relative to the baseline sample.

To quantify typical variation, data from control conditions (resting/non-exercise) were required; however, most of the selected running studies did not include a non-exercise control group, which limited the available data to quantify the typical variation of these markers in resting conditions. For this reason, studies that did not fulfil the exercise intervention criteria but fulfilled the rest of the inclusion criteria (e.g., population, outcomes) and included a control/non-exercise group (with fasted samples collected during the hours and days after baseline) were also used to quantify typical variation.

#### Outcomes and Prioritisation

P1NP and β-CTX-1 were the primary outcomes of interest for this study as these are the reference markers for bone formation and resorption [26].

### Study Design

Any experimental study design that reported the relevant data pre and post a prolonged, continuous running bout or at rest was considered for inclusion, including crossover or parallel group, controlled or uncontrolled, and randomised or non-randomised trials.

### Search Strategy and Study Selection

Studies were identified directly from the list of included articles in a recent systematic review and meta-analysis on the bone (re)modelling marker response to acute exercise interventions [27, 28]. For further details of the protocol, including eligibility criteria, search strategy, study selection and data extraction, of this meta-analysis please refer to Dolan et al. [27]. In summary, seven electronic databases were used to source the material: MEDLINE, Embase, Cochrane CENTRAL, SPORTDiscus, PEDro, LILACS, and IBEC, and were supplemented by citation screening of all selected studies and relevant reviews and book chapters. This search was last updated in May 2022. Additionally, data from a study included in a PhD thesis from the university’s research group that fulfilled the inclusion criteria were included [29].

**Fig. 1:**
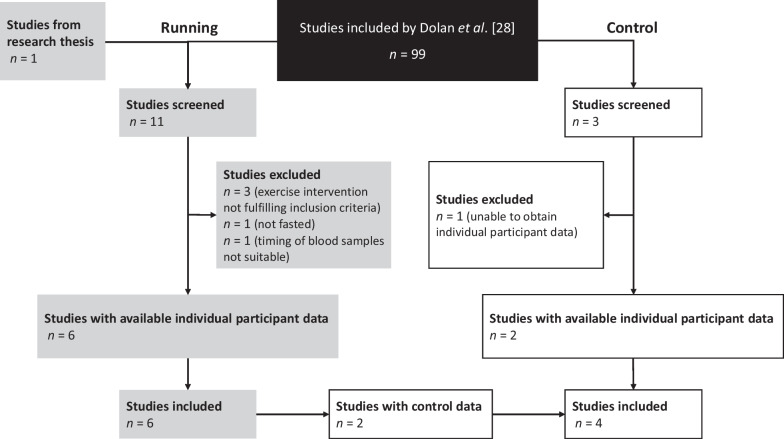
Selection of studies flow diagram. Studies including running data (grey) and studies including control data (white)

The list of articles selected for inclusion in the investigation by Dolan et al. [28] was subsequently screened to identify studies that met the eligibility criteria for the current study. The search strategy and study selection process are illustrated using a modified version of the PRISMA-IPD search flow diagram (Fig. 1).

### Data Extraction and Items

Data from selected studies were first extracted into a custom and pre-piloted spreadsheet (Additional file 1) including: study details (authors; year; study design); participant characteristics (final *n*; training status; age; height; weight; BMI); exercise characteristics (duration; intensity; total work [duration*intensity]); sampling conditions (time of day; diet and exercise standardisation/control before, during and after the intervention, sample handling, assay type); and, if appropriate, intervention group (e.g., trained/recreational participants, placebo).

Anonymised, individual participant raw data were collected from each publication when available (e.g., Additional file 1), or directly from study authors, who were contacted via email, with a maximum of two email attempts made over a period of one month. Individual participant data were entered into codebooks (Additional file 1) and transformed to the same units when included in the codebook (i.e., for P1NP and β-CTX-1 data ng·mL^−1^ was used).

### Risk of Bias Assessment in Individual Studies

The risk of bias for each study was independently assessed in duplicate by two members of the research team using a modified version of the Downs and Black [30] checklist (Additional file 2). This tool was selected because it provides a comprehensive assessment of the methodological quality of both randomised and non-randomised trials in healthcare research and has been validated as a tool to evaluate the quality of reporting as well as internal and external validity [30]. The modified checklist had a total of 16 items, a maximum score of 20 and was tailored to identify the methodological concerns relevant for this analysis. This tool was not used to exclude any eligible studies.

### Statistical Analysis and Calculations

**Fig. 2:**
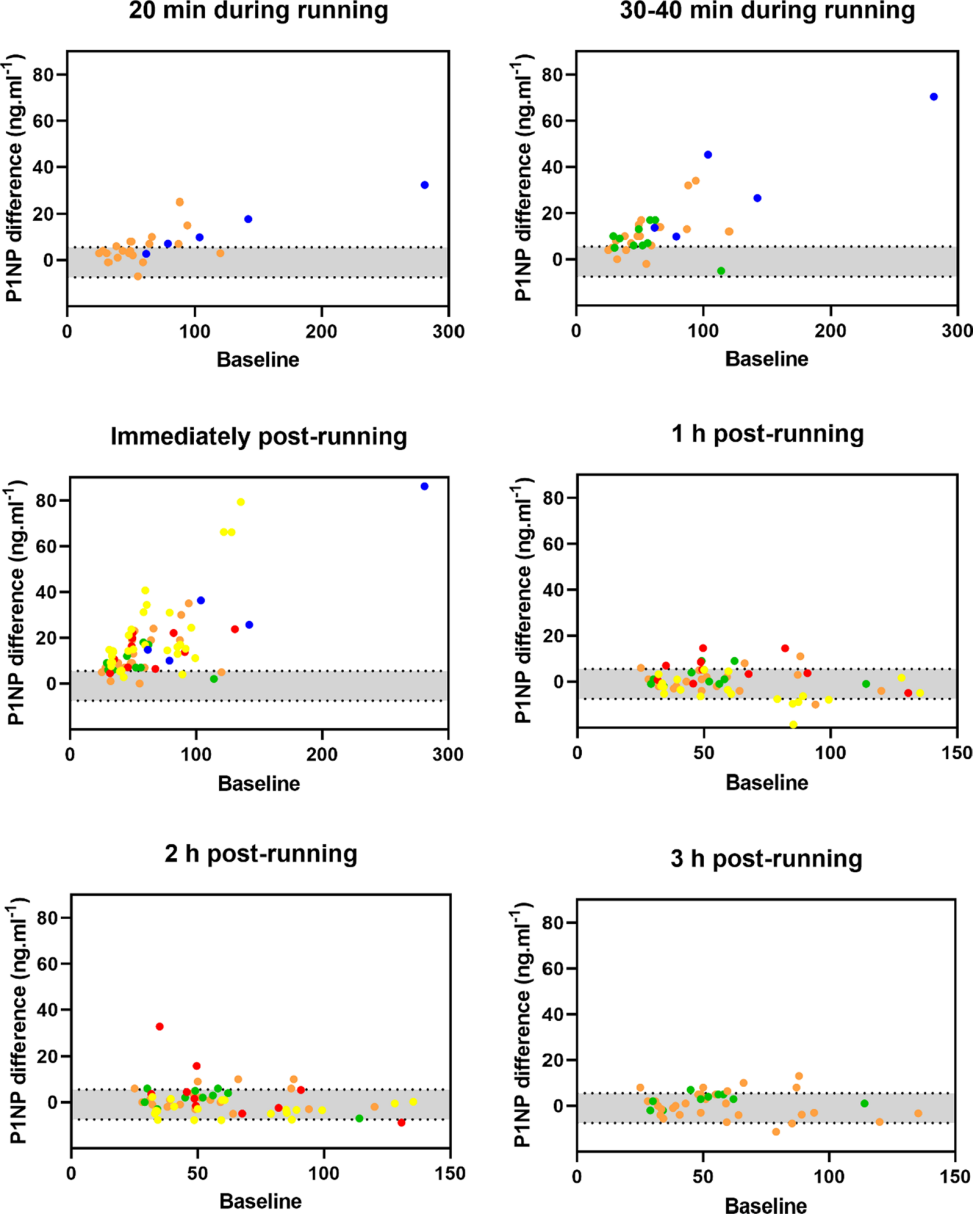
P1NP differences (y axis) from baseline (x axis) during 20 min, 30–40 min, immediately post, 1 h post, 2 h post, and 3 h post a continuous, prolonged running bout. Orange: Scott et al. [33]; blue: Lehrskov et al. [32]; green: Scott et al. [23]; red: Sale et al. [24]; yellow: Townsend et al. [25]. The grey shaded area represents 95% CrI of the mean difference in control conditions (typical hourly variation)

**Fig. 3:**
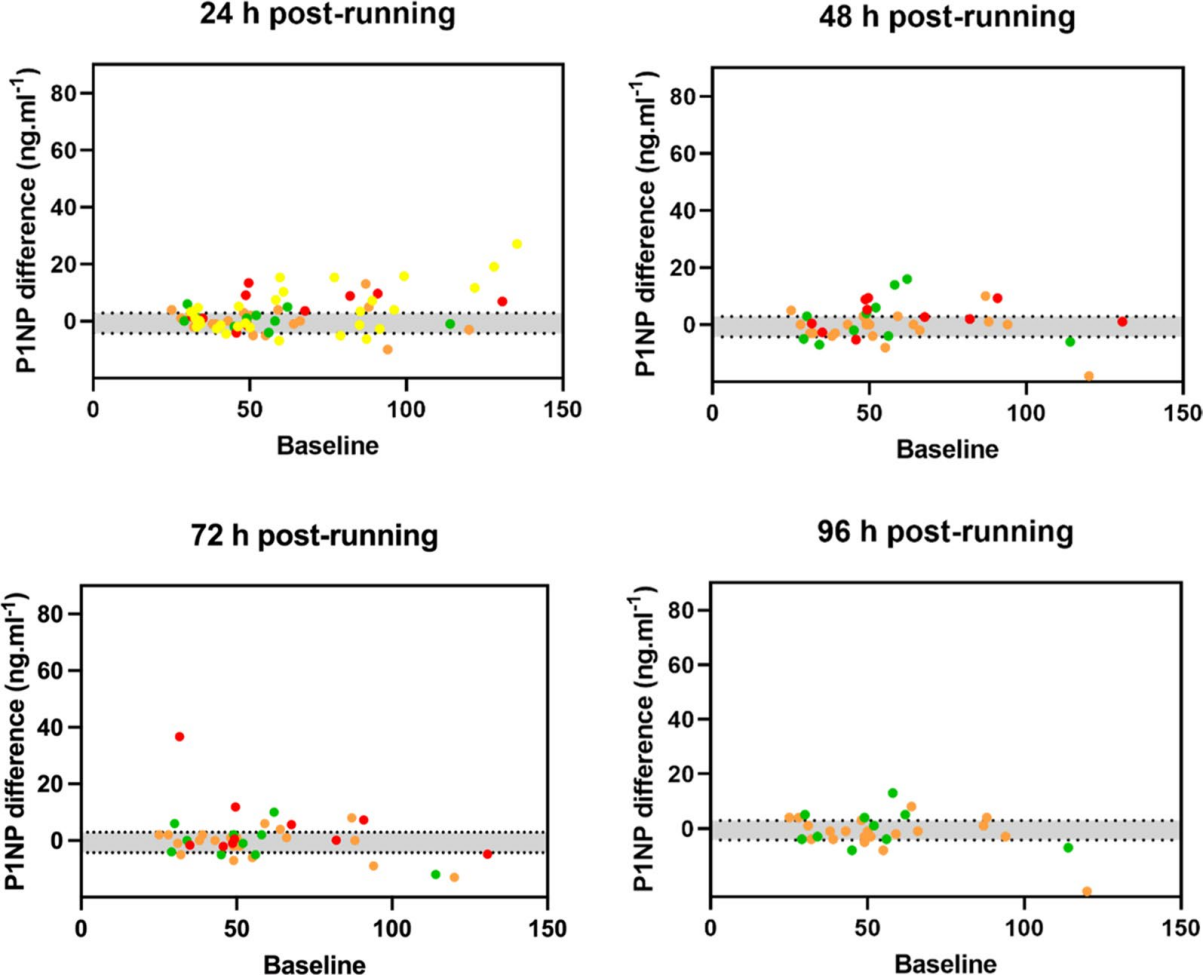
P1NP differences (y axis) from baseline (x axis) 24 h post, 48 h post, 72 h post, and 96 h post a continuous, prolonged running bout. Orange: Scott et al. [33]; green: Scott et al. [23]; red: Sale et al. [24]; yellow: Townsend et al. [25]. The grey shaded area represents 95% CrI of the mean difference in control conditions (typical daily variation)

Individual participant data meta-analyses were conducted to quantify the responses of P1NP and β-CTX-1 for all available time-points during, immediately after, and following exercise. Responses were quantified based upon estimates of the mean difference (by subtracting each time-point from baseline), the standard deviation (SD) of the difference, and the proportion of participants exhibiting an increased response. All models were conducted within a Bayesian framework with random intercepts to account for systematic variation across individual studies. Change scores relative to baseline were calculated for each participant on an absolute scale (ng·mL^−1^) with distributional models used to estimate both the mean difference and standard deviation of the difference. Visual exploration of the data identified the existence of heteroscedasticity, with a positive relationship between baseline values and residuals from change scores. Therefore, the baseline value was entered as a predictor of the standard deviation of the difference. Default priors were used for all parameters, including weakly informative Student-t and half Student-t distributions with 3 degrees of freedom for location and variance parameters (Figs. 2, 3).

Where estimates showed a mean difference and greater standard deviation of change scores for the exercise group, proportion of positive response was estimated by calculating the amount of the distribution (mean difference plus additional standard deviation of the difference) above zero. Inferences from all analyses were performed on posterior samples generated using the Hamiltonian Markov Chain Monte Carlo method (five chains, 100,000 iterations and 50,000 warm-up). Interpretations were based on the median value (0.5-quantile), credible intervals (CrIs) and subjective probabilities calculated from the proportion of the posterior sample that exceeded the relevant value selected. Analyses were performed using the R wrapper package brms [31] interfaced with Stan to perform sampling.

#### Estimations of Typical Variation

With the control data, hourly typical variation of P1NP and β-CTX-1 markers was assessed by estimating the mean difference and SD of difference of blood samples taken at rest before a running bout [23, 32] or in a control group [15] compared to baseline. The typical daily variation of P1NP and β-CTX-1 markers was assessed by estimating the mean difference and SD of difference of blood samples taken 24–96 h post-baseline in control (non-exercise) groups [15, 16] compared to the baseline collected on day 1.

## Results

### Data Collection

#### Running Data

From the selected studies by Dolan et al. [28], five studies were subsequently selected for inclusion in the analysis of the current study [23–25, 32, 33]. One study from a PhD thesis of the university’s group was also included [29]. In total, six studies, with a total of 87 individuals, were included in the analysis for the running data (Table 1). Individual participant data were collected from blood samples measuring P1NP and β-CTX-1 markers at baseline and at all available time-points for each study during and after the running bout (i.e., 20 min during running, 30–40 min during running, 30 min post-running, immediately after, 1 h post-running, 2 h post-running, 3 h post-running, 4 h post-running, 24 h post-running, 48 h post-running, 72 h post-running, and 96 h post-running). For time-points 30 min and 4 h post-running, data were only available from one study and, therefore, were not included in the analyses.

**Table 1:** List of studies included in the analysis

Study	Running/control data	Bone (re)modelling markers	Assay used	Exercise duration/intensity
Evans et al. [15]	Control	β-CTX-1 P1NP	CLIA (IDS) CLIA (IDS)	–
Lehrskov et al. [32]	Running and control	β-CTX-1 P1NP	CLIA (IDS) CLIA (IDS)	60 min at 75% V̇O_2max_
Sale et al. [24]	Running	β-CTX-1 P1NP	ECLIA (Roche) ECLIA (Roche)	120 min at 70% V̇O_2max_
Scott et al. [16]	Control	β-CTX-1 P1NP	ECLIA (Roche) RIA (Orion)	-
Scott et al. [33]	Running	β-CTX-1 P1NP	ECLIA (Roche) RIA (Orion)	60 min at 65% or 75% V̇O_2max_
Scott et al. [23]	Running and control	β-CTX-1 P1NP	ECLIA (Roche) RIA (Orion)	60 min at 65% V̇O_2max_
Townsend et al. [25]	Running	β-CTX-1 P1NP	ECLIA (Roche) ECLIA (Roche)	∼75 min at 75% V̇O_2max_
Varley [29]	Running	β-CTX-1	ELISA (IDS)	120 min at 70% V̇O_2max_

*CLIA* chemiluminescence immunoassay; *ECLIA* electro-chemiluminescence assay; *ELISA* Enzyme-linked immunosorbent assay; *P1NP* amino-terminal propeptide of type 1 procollagen; *RIA* radioimmunoassay; *β-CTX-1* carboxy-terminal telopeptide of type 1 collagen

#### Control Data

From the six studies included for the running data, two [23, 32] collected blood samples (i.e., 1–3 samples) in resting (control) conditions before the running bout. Three additional studies [15–17], which fulfilled all inclusion criteria except the intervention characteristics, but included a non-exercise control group were also identified. However, individual participant data were only obtained, and thereby included, from two of these studies [15, 16] for the control data (Table 1). For these four studies [15, 16, 23, 32], individual participant data were obtained from blood samples collected at baseline and during a 1–2.5 h period (hourly) and 24–96 h (daily) after the baseline sample in the control conditions/group. These data were used to estimate the hourly and daily P1NP and β-CTX-1 mean difference and SD of the difference in control (resting) conditions.

### Typical Hourly and Daily Variation of P1NP and β-CTX-1

The typical hourly and daily variation in P1NP and β-CTX-1 was determined by the mean difference and SD of the difference in control conditions (Table 2). There was limited evidence of a mean difference for hourly (0.06 [95% CrI − 7.5 to 5.5] ng·mL^−1^) and daily (-0.39 [95% CrI − 4.3 to 2.9] ng·mL^−1^) P1NP changes based on median estimates being close to zero and wide CrIs. Slightly higher variation in the hourly changes (± 7.6 [95% CrI 6.8 to 8.5] ng·mL^−1^) was estimated compared with the daily changes (± 5.7 [95% CrI 5.1 to 6.5] ng·mL^−1^). Stronger evidence was obtained for a mean difference for hourly β-CTX-1 changes, with the median and majority of the CrI indicating a decrease (-0.13 [95% CrI − 0.34 to 0.06] ng·mL^−1^). A wide CrI with median close to zero provided limited evidence of a mean difference for daily β-CTX-1 changes (-0.03 [95% CrI − 0.54 to 0.30] ng·mL^−1^). The SD of the difference was consistent between hourly (± 0.11 [95% CrI 0.11 to 0.12] ng·mL^−1^) and daily (± 0.10 [95% CrI 0.09 to 0.11] ng·mL^−1^) changes of β-CTX-1. There was consistent evidence of heteroscedasticity with greater change score magnitudes for those with higher baselines.

**Table 2:** Hourly and daily typical variation of P1NP and β-CTX-1 in control conditions

Marker	Studies	Number of participants and observations	Mean difference [95% CrI]	SD of difference [95% CrI]
Hourly				
P1NP (ng·mL^−1^)	Lehrskov et al. [32], Scott et al. [23], Evans et al. [15]	Participants *n* = 27 Observations *n* = 58	0.06 [− 7.5 to 5.5]	7.6 [6.8 to 8.5]^a^
β-CTX-1 (ng·mL^−1^)	Lehrskov et al. [32], Scott et al. [23], Evans et al. [15]	Participants *n* = 27 Observations *n* = 58	− 0.13 [− 0.34 to 0.06]	0.11 [0.11 to 0.12]^a^
Daily				
P1NP (ng·mL^−1^)	Evans et al. [15], Scott et al. [16]	Participants *n* = 22 Observations *n* = 52	− 0.39 [− 4.3 to 2.9]	5.7 [5.1 to 6.5]^a^
β-CTX-1 (ng·mL^−1^)	Evans et al. [15], Scott et al. [16]	Participants *n* = 22 Observations *n* = 52	− 0.03 [− 0.54 to 0.30]	0.10 [0.09 to 0.11]^a^

^a^Evidence of heteroscedasticity

### P1NP and β-CTX-1 Responses to a Prolonged, Continuous Running Bout

#### Bone Formation

In contrast to the control condition (0.06 [95% CrI − 7.5 to 5.5] ng·mL^−1^), there was clear evidence that the levels of circulating P1NP increased during and immediately after the running bout with mean differences of 4.2 [95% CrI 0.2 to 8.8] ng·mL^−1^ at 20 min during the running bout, 9.2 [95% CrI 5.3 to 14.3] ng·mL^−1^ at 30–40 min during the running bout, and 12.0 [95% CrI 8.4 to 16.0] ng·mL^−1^ immediately after the running bout (Table 3). Greater SD of the difference was identified only at 30–40 min during (± 8.1 [95% CrI 7.1 to 9.4] ng·mL^−1^) and immediately (± 10.2 [95% CrI 9.3 to 11.3] ng·mL^−1^) after the running bout (Table 3) compared to the typical hourly variation (± 7.6 [95% CrI 6.8 to 8.5] ng·mL^−1^ (Table 2). For these three time-points (20 min during, 30–40 min during and immediately after) the proportion of response was estimated as close to 100% (Table 3), indicating that close to all participants would be expected to demonstrate an increase in P1NP levels. From one hour after finishing the running bout and for the next three hours, P1NP returned to “normal” levels, with comparable mean differences and SD of difference (Table 3) than the typical hourly variation (Table 2). Likewise, for the four days (24–96 h) after the baseline in the running conditions, P1NP mean differences and SD of the difference (Table 3) were comparable with the typical daily variation (Table 2). The proportion of response was not estimated for these time-points due to these similarities and, therefore, there was a lack of evidence of inter-individual response. There was evidence of heteroscedasticity across all time-points except at 2 h and 72 h post-running.

**Table 3:** Responses of P1NP bone formation marker to a prolonged, continuous running bout

P1NP (ng·mL^−1^)	Mean difference [95% CrI]	SD of difference [95% CrI]	*P* of increased variation	Proportion of response
Hourly				
20 min during running (25 observations / 2 studies)	4.2 [0.2 to 8.8]	6.1 [5.2 to 7.3]^a^	0.088	1.0 75% CrI [0.99 to 1.0]
30–40 during running (35 observations / 3 studies)	9.2 [5.3 to 14.3]	8.1 [7.1 to 9.4]^a^	0.700	1.0 75% CrI [0.99 to 1.0]
Immediately after (75 observations / 5 studies)	12.0 [8.4 to 16.0]	10.2 [9.3 to 11.3]^a^	> 0.999	1.0 75% CrI [0.90 to 1.0]
1 h post-running (75 observations / 5 studies)	1.1 [− 3.1 to 5.2]	5.0 [4.5 to 5.6]^a^	< 0.001	–
2 h post-running (60 observations / 4 studies)	0.6 [− 3.1 to 4.3]	6.1 [5.5 to 6.8]	0.016	–
3 h post-running (40 observations / 3 studies)	0.6 [− 4.6 to 5.7]	4.6 [3.8 to 5.1]^a^	< 0.001	–
Daily				
24 h post-running (70 observations / 4 studies)	1.4 [− 0.5 to 3.5]	5.2 [4.8 to 5.8]^a^	0.172	–
48 h post-running (40 observations / 3 studies)	0.6 [− 2.3 to 3.7]	5.9 [5.2 to 7.1]^a^	0.612	–
72 h post-running (40 observations / 3 studies)	0.5 [− 2.8 to 3.9]	7.8 [6.9 to 9.0]	0.999	–
96 h post-running (30 observations / 2 studies)	− 0.4 [− 3.9 to 3.0]	5.4 [4.7 to 6.4]^a^	0.349	–

^a^Evidence of heteroscedasticity. Proportion of response was only calculated where there was strong evidence of a mean difference

#### Bone Resorption

Although β-CTX-1 blood levels showed a small decrease in mean differences during the running bout and for the four hours after finishing the running bout, the mean and SD of the differences (Table 4) were similar to the β-CTX-1 typical hourly variation (-0.13 ± 0.11 ng·mL^−1^) (Table 2). For the four days (24–96 h) after the baseline in the running conditions, the distribution of the β-CTX-1 differences (Table 4) were similar to the β-CTX-1 typical daily variation (-0.03 ± 0.10 ng·mL^−1^ (Table 2). The proportion of response was not estimated for any time-points due to the small mean differences in the running conditions and the similarities in the SD of the difference between running and control conditions. There was evidence of heteroscedasticity across all time-points except for 72 h and 96 h post-running time-points.

**Table 4:** Responses of β-CTX-1 bone resorption marker to a prolonged, continuous running bout

β-CTX-1 (ng·mL^−1^)	Mean difference [95% CrI]	SD of difference [95% CrI]	*P* of increased variation	Proportion of response
Hourly				
20 min during running (25 observations / 2 studies)	− 0.09 [− 0.55 to 0.37]	0.04 [0.03 to 0.05]^a^	< 0.001	–
30–40 during running (35 observations / 3 studies)	− 0.06 [− 0.29 to 0.14]	0.10 [0.08 to 0.11]^a^	0.563	–
Immediately after (116 observations / 6 studies)	− 0.01 [− 0.09 to 0.06]	0.13 [0.12 to 0.14]^a^	0.996	–
1 h post-running (60 observations / 4 studies)	− 0.02 [− 0.13 to 0.08]	0.10 [0.09 to 0.12]^a^	0.244	–
2 h post-running (60 observations / 4 studies)	− 0.08 [− 0.18 to 0.01]	0.10 [0.09 to 0.11]^a^	0.109	–
3 h post-running (40 observations / 3 studies)	− 0.13 [− 0.36 to 0.03]	0.11 [0.10 to 0.13]^a^	0.576	–
Daily				
24 h post-running (111 observations / 5 studies)	0.01 [− 0.02 to 0.04]	0.12 [0.11 to 0.13]^a^	> 0.999	–
48 h post-running (81 observations / 4 studies)	0.01 [− 0.06 to 0.07]	0.15 [0.14 to 0.16]^a^	> 0.999	–
72 h post-running (81 observations / 4 studies)	0.06 [− 0.09 to 0.19]	0.32 [0.29 to 0.35]	> 0.999	–
96 h post-running (30 observations / 2 studies)	− 0.03 [− 0.27 to 0.24]	0.09 [0.08 to 0.11]	0.311	–

^a^Evidence of heteroscedasticity. Proportion of response was only calculated where there was strong evidence of a mean difference

### Risk of Bias Assessment

The application of the modified Downs and Black [30] checklist (Additional file 2) resulted in the classification of seven studies as high quality and two studies as moderate quality (Table 5). The most common reasons why studies were downgraded were because of lack of details provided regarding the storage and handling of the blood samples [15, 16], the lack of specification of the standardisation of the exact time of the day when the fasted morning baseline was collected [15, 32], and the inadequate or absent standardisation/monitoring of important nutrition and diet variables [15, 29, 32]. All running studies [23–25, 29, 32, 33] were downgraded because the during and post running data were not corrected for shifts in plasma volume.

**Table 5:** Risk of bias in individual studies

Study	Score	Quality
Evans et al. [15]	17/20	
Lehrskov et al. [32]	16/20	
Sale et al. [24]	19/20	
Scott et al. [16]	19/20	
Scott et al. [33]	18/20	
Scott et al. [23]	19/20	
Townsend et al. [25]	19/20	
Varley [29]	18/20	

Green circle, high quality; yellow circle, moderate quality

## Discussion

The key findings of the study were that: (i) P1NP increased exclusively during and immediately after running, and there was a lack of evidence of changes in β-CTX-1 linked to running, (ii) the inter-individual variability of P1NP and β-CTX-1 change scores were similar between resting (control) conditions and during and after running, except for P1NP levels during and immediately after the running bout, and, therefore, (iii) there was an overall lack of inter-individual response in P1NP and β-CTX-l linked to running, with reported decreases in β-CTX during the hours after running not being attributable to the running intervention.

Increases in P1NP levels were limited to during and immediately after exercise, and this consistently occurred within all participants (estimated proportion of response ~ 100%). Because these changes were sudden and transient, however, it seems unlikely that they reflect any meaningful increase in bone formation. Similar results were reported in the recent meta-analysis by Dolan et al. [28], where they pooled the acute responses of bone (re)modelling markers after different exercise interventions, showing increases in P1NP within 15 min of the cessation of exercise. It is possible that this transient increase in circulating P1NP could be due to leakage of P1NP from the connective tissue into the circulation or due to haemodynamic shifts. P1NP is not a bone-specific marker and can be affected by the metabolism of collagen from other tissues [26]. Although fluids were provided during the running bout in two studies [24, 33], shifts in plasma volume were not accounted for in any of the running studies included in this review, which could contribute toward explaining the transiently higher concentrations. Brahm et al. [34] reported similar, sudden and transient, increases in C-terminal propeptide of type 1 procollagen (P1CP) in young individuals after a running to exhaustion intervention with a total duration of ~ 35 min, which mirrored changes in plasma volume (showing decreases following the same pattern) and corresponded to increases in haematocrit. They reported no significant changes in P1CP when correcting for plasma volume shifts [34]. As such, it seems plausible that the increases observed herein are due to biological artefact, rather than representing an actual increase in bone formative processes.

The stimulation of bone formation in response to exercise may require a longer period, although how long exactly may be required is currently unknown. Our statistical model based on available data led to the conclusion of no change in P1NP levels in the 1–3 h and 1–4 days post-running, which indicates that a single prolonged, continuous running bout did not stimulate bone formation, at least up until the fourth day after the running bout. Most studies that have measured bone (re)modelling markers after an acute exercise intervention have only done so for a few days (1–3 days) after the intervention [35–40] and, therefore, there are no data available on longer-term changes of bone formation markers in response to a single exercise session. In contrast, longitudinal studies in healthy adult populations evaluating at the chronic responses of bone (re)modelling markers to repeated exercise training of various types have consistently shown increased resting levels of bone formation markers, including P1NP [41–50]. Therefore, bone formation responses measured by changes in P1NP levels might take longer than the 4 days used in studies thus far.

For circulating levels of β-CTX-1, despite showing small decreases during and in the hours after running, there is no evidence that these changes were caused by the running intervention, given that they were similar in magnitude to the reductions shown in the non-exercise control data. Together, these results suggest that the small decreases in circulating β-CTX-1 shown during and in the hours after running were caused by measurement error rather than as a result of the running intervention. These reductions in β-CTX-1 coincide with the circadian rhythm of this biomarker under fasting conditions [14], peaking in the early morning and declining in the later morning hours [51, 52].

Furthermore, aggregate meta-analytic evidence [28] suggests that β-CTX-1 responses to exercise are influenced by the type of exercise, with moderate to large increases shown from 15 min to 2 h after long-duration cycling. Increases in β-CTX-1 could be explained by increases in parathyroid hormone (PTH), triggered by reductions in serum calcium, that subsequently stimulates osteoclastic bone resorption [53]. Although this mechanism seems to agree with the β-CTX-1 increases with cycling interventions [54], it does not explain the lack of a response reported herein, given that increased PTH has also been observed in response to similar running bouts as were investigated herein [23–25, 33].

For the 1–4 days after the running bout, β-CTX-1 blood levels were also similar to the daily typical variation determined by the control data, indicating that the running intervention did not result in significant responses to β-CTX-1 circulating levels. Similar results were reported by the Dolan et al. [28] meta-analysis, which included studies with different designs and exercise interventions, although they showed some evidence of increases in β-CTX-1 at 72 h post-exercise. The lack of a response (i.e., increase) in β-CTX-1 shown herein, could be considered as a beneficial outcome for bone adaptations if it is interpreted as the lack of resorption activity that can lead to bone loss. In contrast, the initial increase in bone resorption markers could be indicative of the activation of the bone (re)modelling cycle [55]. In this case, it could be concluded that a single running bout does not stimulate bone remodelling, at least within the next four days. Bone (re)modelling is, however, a nuanced process that is continuously ongoing at different stages across different skeletal sites and site-specific bone adaptations to exercise interventions might not be reflected in systemic bone (re)modelling markers.

### Strengths and Limitations

Studies included in this meta-analysis were classified as high quality (*n* = 8) overall. It should be noted, however, that the inclusion criteria applied herein were thorough and delimited, meaning low quality studies would likely not have met this criterion. In the Dolan et al. [28] meta-analysis, which had less restricted inclusion criteria and included a larger number of studies (*n* = 99), the general quality of the studies was reported as moderate. While more inclusive criteria would have allowed the inclusion of a greater number of studies and, thereby, more data points, this would have also added more variability. The aim of this meta-analysis was to investigate the responses of P1NP and β-CTX-1 in very specific conditions by reducing potential sources of variability, such as the type of exercise intervention (i.e., impact level, duration, and intensity, intermittent/continuous), participant characteristics (i.e., age, sex, and health status) and study design (i.e., feeding/fasting conditions, time of the day). Removing these sources of variability allowed for a better understanding of the inter-individual variability caused by factors external to the intervention itself, such as circadian variation.

This systematic review with individual participant data meta-analysis was not without limitations, including those inherited from the included studies. Although all included studies collected a baseline sample in the morning, the exact time of the day when the fasted baseline sample was taken only varied from 0800–0840 but was not specified in two studies [15, 32]. Similarly, the exact time of the day when the running bout began was different across studies. These factors could have impacted the changes in bone (re)modelling markers; particularly β-CTX-1, which has a more pronounced variation due to its circadian rhythm [14]. Additionally, habitual dietary and nutritional factors, such as energy availability, macronutrient composition of the diet and vitamin D and calcium intakes, were not controlled in the included studies, and could have affected P1NP and β-CTX-1 baseline levels (for a review please see [56]). The baseline level of a marker might be an important variable determining the subsequent response to exercise, and potentially to other interventions as well, as the heteroscedasticity shown in the participant data of this meta-analysis suggested that those participants with higher baselines had greater changes in both markers and across time-points, which requires further investigation.

**Fig. 4:**
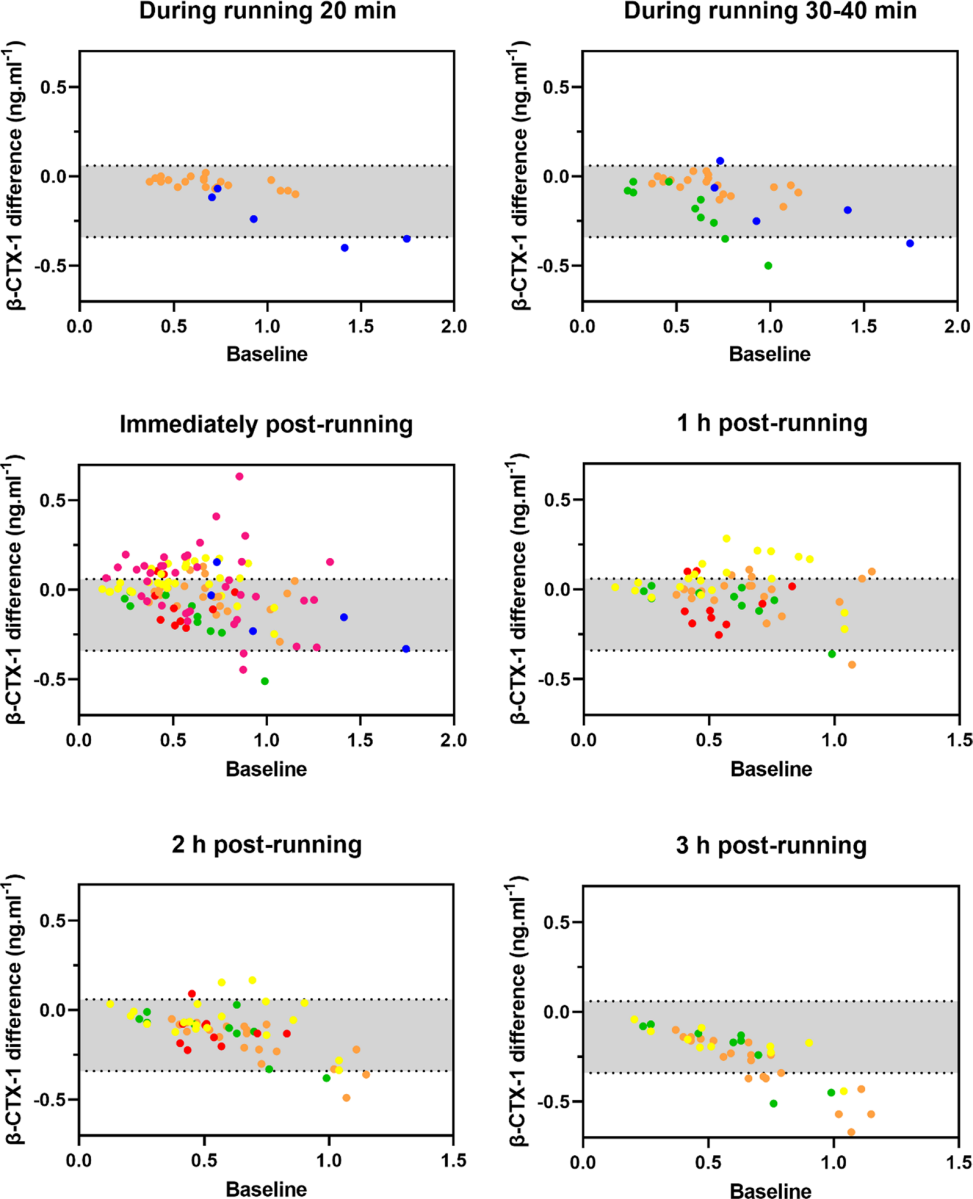
β-CTX-1 differences (y axis) from baseline (x axis) during 20 min, 30–40 min, immediately post, 1 h post, 2 h post, and 3 h post a continuous, prolonged running bout. Orange: Scott et al. [33]; blue: Lehrskov et al. [32]; green: Scott et al. [23]; red: Sale et al. [24]; yellow: Townsend et al. [25] pink: Varley [29]. The grey shaded area represents 95% CrI of mean difference in control

**Fig. 5:**
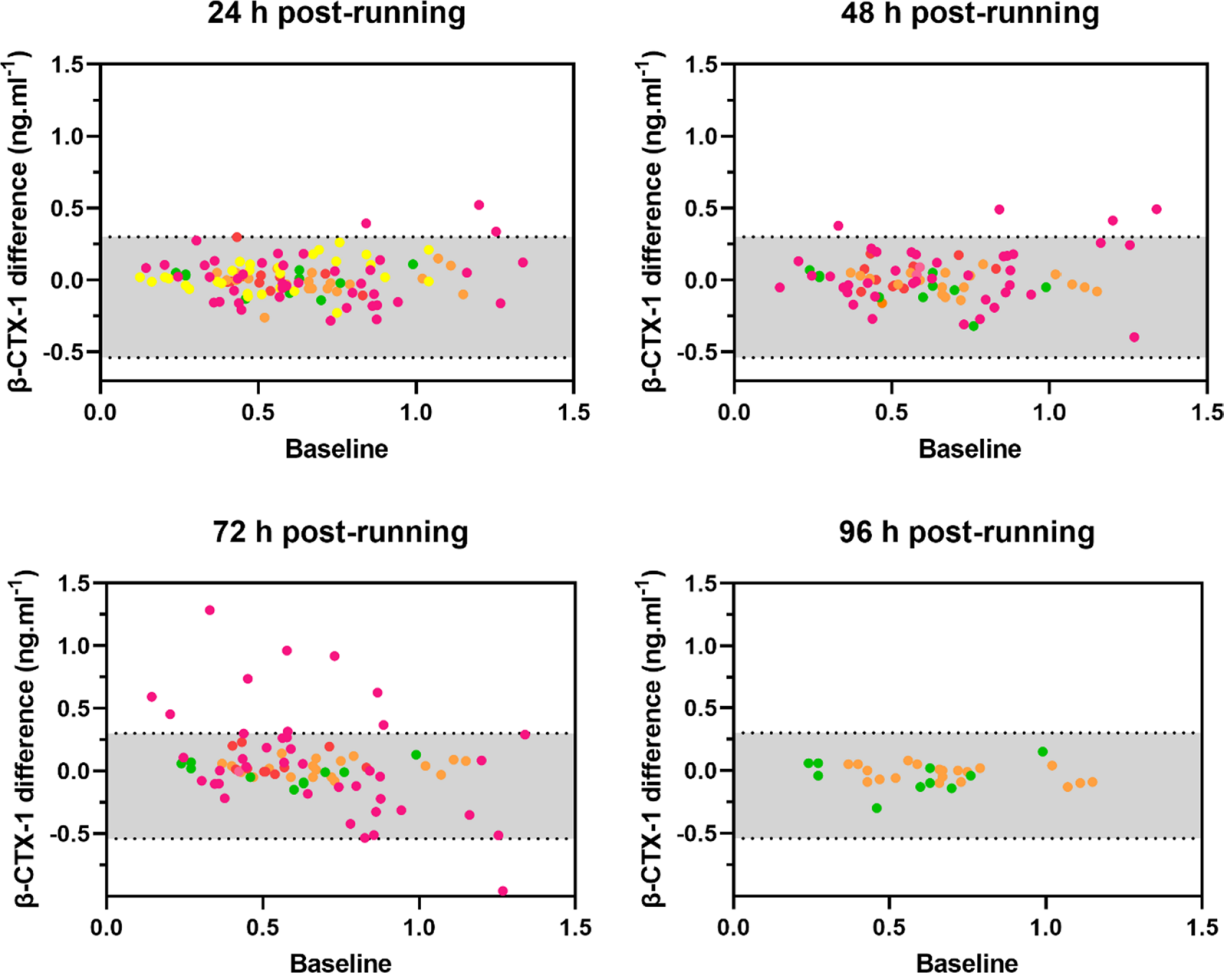
β-CTX-1 differences (y axis) from baseline (x axis) 24 h post, 48 h post, 72 h post, and 96 h post a continuous, prolonged running bout. Orange: Scott et al. [33]; green: Scott et al. [23]; red: Sale et al. [24]; yellow: Townsend et al. [25]; pink: Varley [29]. The grey shaded area represents 95% CrI of mean difference in control conditions (typical daily variation)

Only two studies included a non-exercise control group, which means that the control data used to estimate the typical variation of P1NP and β-CTX-1 in resting conditions were predominantly from different participants (although with similar characteristics). It is possible that the inter-individual variability was greater than if all participant control data had been obtained from the same running participants. Nonetheless, the mean differences and variability (i.e., SD of the difference) in P1NP and β-CTX-1 were similar between the control and running data. For the running data, the running interventions of the included studies had a relatively limited range of durations and intensities, which could have added variability in our results. While exercise duration, intensity, and total work done might modulate bone (re)modelling markers responses [28, 33], the consistency and relevance of these effects are unclear from the current evidence. Nonetheless, the mean differences and variability (i.e., SD of the difference) in P1NP and β-CTX-1 were similar between the control and running data. Another factor that could have increased variability in all data is the measurement error from the instrumentation (e.g., variation of analytical assays used to measure bone biomarkers in the included studies). Various types of assays were used across studies, which have different intra- and inter-assay coefficients of variation, generally ranging from 1.4–4.9% (P1NP) and 2.1–5.3% (β-CTX-1) [57]. This variation can be critical for exercise research given the overall small responses that bone (re)modelling markers exhibit after acute exercise interventions [28]. For example, β-CTX-1 samples analysed using ELISA methods, as in the Varley [29] study, seemed to yield higher variability in the data (Figs. 4 and 5, pink dots) compared to others.

The current meta-analysis only included studies with a running intervention in young healthy adult males because similar studies in female populations are lacking. Available literature on P1NP and/or β-CTX-1 responses to exercise in females usually involves post-menopausal populations [40, 58, 59]. Only a limited number of studies have investigated the acute responses of reference markers P1NP and/or β-CTX-1 to exercise (e.g., jogging, brisk walking with resistance training, football) in young females [40, 60, 61]. No studies have directly compared these responses between males and females; however, in their meta-analysis, Dolan and colleagues [28] showed that sex did not influence exercise-associated changes of markers P1NP and β-CTX-1. In addition, studies in older male populations in this area are also lacking, and older adults might have different β-CTX-1 responses to aerobic exercise as highlighted in a recent systematic review [62]; hence, results from the current analysis might not translate to older populations.

### Implications for Future Research

The majority of studies included in this meta-analysis did not include a control (non-exercise) group because they were designed to investigate how various factors (e.g., nutrition, exercise intensity) may moderate the bone (re)modelling marker responses to a running bout. Indeed, this study design is commonly used within exercise research and only about a quarter of studies assessing acute exercise responses of bone (re)modelling markers included a control group [28]. It is recommended that non-exercise control groups are included in future studies to quantify the variability of the instrumentation noise (i.e., from assays) and biological noise (e.g., from circadian rhythms) [18], and to establish if exercise interventions of different kinds produce an effect on bone (re)modelling markers.

Bone (re)modelling markers have not yet been validated or linked to a primary reference measurement because there is no alternative reference measurement system available that can act as a higher order standard or gold standard [57], and it is not clear whether they can predict changes detected by imaging techniques, such as dual-energy X-ray absorptiometry (DXA) or peripheral quantitative computed tomography (pQCT) [26]. Bone (re)modelling markers are systemic and do not necessarily represent local bone adaptations/changes. Therefore, studies utilising bone (re)modelling markers to investigate the bone responses to acute or short-term exercise interventions will likely be missing key information about the local effects that loading has on the skeleton and they need to be interpreted with understanding of this limitation.

It is important that studies including bone (re)modelling markers adhere to the recommended standardisation guidelines [26, 57, 63], control important factors before the intervention (e.g., nutrition, sleep, physical activity), clearly report the time of the day of all measures, sampling timing, storage, and handling of the samples, and report assay quality control information, which would reduce inter-individual variability and help when making comparisons with other studies. Given the potentially misleading increases in P1NP during and immediately post-running reported herein, studies should also consider shifts in plasma volume and fluid lost or report both adjusted and unadjusted data for changes in plasma volume.

###  Summary and Conclusions

This individual participant data meta-analysis determined that a prolonged, continuous bout of treadmill running (60–120 min at 65–75% V̇O_2max_) does not result in changes in bone (re)modelling, as determined by P1NP and β-CTX-1, in young healthy adult males. Whilst there was evidence of a transient increase of P1NP during and immediately after running, this response was likely caused by biological aspects (e.g., shifts in plasma volume, leakage from other connective tissues) rather than being reflective of bone formation. Similar small decreases in β-CTX-1 were shown in control and running data, suggesting that these changes were due to the marker’s circadian rhythm and not the running intervention. Hence, it remains unclear whether a single running bout produces bone adaptations, but indirect bone (re)modelling markers P1NP and β-CTX-1 markers failed to capture any potential responses. There is a need for individual studies that investigate the acute responses of bone (re)modelling markers to different types of exercise and across different populations, which include a control (non-exercise) group.

### Supplementary Information

**Additional file 1**. Data extraction spreadsheet and codebooks.

**Additional file 2**. Modified Downs & Black checklist.

## Data Availability

Data are available on request from the authors.

## Abbreviations

BMD: Bone mineral density
CLIA: Chemiluminescence immunoassay
CrI: Credible intervals
DXA: Dual-energy X-ray absorptiometry
ECLIA: Electro-chemiluminescence assay
ELISA: Enzyme-linked immunosorbent assay
IPD: Individual participant data
P1NP: N-terminal propeptide of type 1 procollagen
PICOS: Population, Intervention, Comparator, Outcomes and Study Design
pQCT: Peripheral quantitative computed tomography
PRISMA: Preferred Reporting Items for Systematic Review and Meta-Analysis
PTH: Parathyroid hormone
RIA: Radioimmunoassay
SD: Standard deviation
V̇O_2max_: Maximum rate of oxygen consumption
β-CTX-1: C-terminal telopeptide of type 1 collagen
