# Supplementary material for: P1NP and β-CTX-1 Responses to a Prolonged, Continuous Running Bout in Young Healthy Adult Males: A Systematic Review with Individual Participant Data Meta-analysis
Source: Sports Med Open. 2023 Sep 19;9:85. doi: 10.1186/s40798-023-00628-x (PMC10509102; doi:10.1186/s40798-023-00628-x)
Supplement: Supplementary file 2 — Additional file 2. Modified Downs & Black checklist. [file 40798_2023_628_MOESM2_ESM.docx]

**P1NP and β-CTX-1 responses to a prolonged, continuous running bout in young healthy adult males: a systematic review with individual participant data meta-analysis**

Supplementary File 2

Modified Downs & Black checklist

**Q.1.** *Is the hypothesis/aim/objective of the study clearly described*? Yes = 1; No = 0

**Q.2.** *Are the main outcomes to be measured clearly described in the introduction or methods section?* If the main outcomes are first mentioned in the results section, answer no. Yes = 1; No = 0.

**Q.3.** *Are the characteristics (e.g., age, height, weight, training and health status) and the inclusion/exclusion criteria (e.g., non-smokers, injury free for the previous 12 months, not taking any medication, or suffering from any condition known to affect bone metabolism) of the participants included in the study clearly described?* Yes = 2 (characteristics AND inclusion/exclusion criteria); Yes = 1 (characteristics OR inclusion/exclusion criteria); No = 0.

**Q.4.** *Are the characteristics of the exercise bout clearly described?* Including, type, intensity and duration should be described. For control studies, resting should be confirmed. If they provide a nutritional supplement the exact type and dose should be provided. Yes = 1, No = 0.

**Q.5.** *Does the study provide estimates of the random variability in the data for the main outcomes?* In non-normal data, inter-quartile range should be reported. In normal data, standard deviation, standard error or confidence intervals should be reported. Yes = 1; No = 0.

**Q.6.** *Are the main results of the study clearly described?* Simple outcome data should be reported (including tables and figures) for all major results so the reader can check the major analyses and conclusions. This does not cover statistical tests. Yes = 1; No = 0.

**Q.7.** *Were the interventions approved by an ethics committee?* Answer yes if they confirm they have ethical approval Yes = 1; No = 0.

**Q.8.** *If any of the results of the study were based on ‘data dredging’ was this made clear?* Any analyses that had not been planned at the outset should be clearly indicated. If no retrospective subgroup analyses were reported, then answer yes. Yes = 1; No = 0; Unable to determine = 0.

**Q.9.** *Was the timing of blood sampling clearly described?* Answer yes if the precise time-points were provided. Answer no if it is not clear exactly when the blood samples were drawn during the trial. Yes = 1; No = 0; Unable to determine = 0.

**Q.10.** *Were the laboratory methodologies (e.g., blood samples storage and handling, CV of assays used) appropriately described?* Yes = 2 (information about sample storage and handling, AND assays CV is provided); Yes = 1 (information about sample storage and handling, OR assays’ CV is provided); No = 0; Unable to determine = 0.

**Q.11.** Were study participants randomised to intervention groups or was the order of trials randomised if using *a crossover design?* Answer yes if the order of trials, treatment, or allocation to groups, was randomly assigned. If it was not possible for the study to be randomised (*e.g*., single-trial studies) or if the intervention of interest was not exercise answer yes. Yes = 1; No = 0; Unable to determine = 0.

**Q.12.** *Were multiple trials separated by a clear washout period?* Answer yes if there was a washout period between multiple trials in crossover/counterbalanced designs. For studies with different designs (*e.g*., single-trial, randomised groups) answer yes. Yes = 1; No = 0; Unable to determine = 0.

**Q.13.** *Was at least one familiarization session conducted prior to exercise testing?* Answer yes if they conducted a familiarization exercise trial, **or if familiarization was not necessary** (*e.g.,* if the study uses a single or **non-performance-based** exercise bout, and control/resting interventions). Yes = 1; No = 0; Unable to determine = 0.

**Q.14.** *Were the exercise test conditions adequately standardised and described?* Factors to consider include confirmation of the time of day that testing was conducted (score yes if the exact time of day that tests were conducted was reported, and this was the same for all participants), and control for unusual activity (score yes if they requested that participants avoid unusual or very strenuous activity for at least 24 hours prior to the test) or nutritional factors in the days prior to the exercise test (score yes if they request participants to maintain usual feeding habits the day before). Yes (all 3 factors considered) = 3; Yes (most factors (2 of the 3) considered) = 2; Yes (some (1 of the 3) factors considered) = 1; No = 0; Unable to determine = 0.

**Q.15.** *Was fasting status for baseline blood sampling confirmed*? Answer yes if the strategy for standardization is indicated as an overnight fast. Yes = 1, No = 0, Unable to determine = 0.

**Q.16.** Were samples corrected for plasma volume changes *or appropriately accounted for with fluid intake*? Answer yes for resting/control studies. Yes = 1; No = 0; Unable to determine = 0

Max attainable score = 20. The combined score will be used to categorise each study according to 4 categories, i.e., High (18 - 20), Moderate (15 - 17), Low (11 - 14) or Very Low (≤ 10)

Note: For any question where the response cannot be ascertained based on the information presented in the article, score 0 (unable to tell).

**Original Reference:**

Downs, S.H., & Black, N. The feasibility of creating a checklist for the assessment of the methodological quality both of randomised and non-randomised studies of health care interventions. (1998). *Journal of Epidemiology and Community Health*, 52(6), pp.377–384. https://doi.org/10.1136/jech.52.6.377
